# Identification of TEFM as a potential therapeutic target for LUAD treatment

**DOI:** 10.1186/s12967-024-05483-2

**Published:** 2024-07-29

**Authors:** Wenxuan Hu, Jian Yang, Kang Hu, Gaomeng Luo, Zhike Chen, Zihao Lu, Yongsen Li, Xin Lv, Jun zhao, Chun Xu

**Affiliations:** 1 Institute of Thoracic Surgery, The First Affiliated Hospital of Soochow University, Suzhou, China; 2 Department of Thoracic Surgery, The First Affiliated Hospital of Soochow University, Suzhou, China

**Keywords:** TEFM, NSCLC, Mitochondria

## Abstract

**Background:**

Molecularly targeted therapies have recently become a hotspot in the treatment of LUAD, with ongoing efforts to identify new effective targets due to individual variability. Among these potential targets, the mitochondrial transcription elongation factor (TEFM) stands out as a crucial molecule involved in mitochondrial synthetic transcriptional processing. Dysregulation of TEFM has been implicated in the development of various diseases; however, its specific role in LUAD remains unclear.

**Methods:**

We conducted a comprehensive analysis of TEFM expression in LUAD, leveraging data from the TCGA database. Subsequently, we validated these findings using clinical specimens obtained from the First Affiliated Hospital of Soochow University, employing western blotting and qRT-PCR techniques. Further experimental validation was performed through the transfection of cells with TEFM overexpression, knockdown, and knockout lentiviruses. The effects of TEFM on LUAD were evaluated both in vitro and in vivo using a range of assays, including CCK-8, colony formation, EdU incorporation, Transwell migration, Tunel assay, flow cytometry, JC-1 staining, and xenograft tumour models.

**Results:**

Our investigation uncovered that TEFM exhibited elevated expression levels in LUAD and exhibited co-localization with mitochondria. Overexpression of TEFM facilitated malignant processes in LUAD cells, whereas its silencing notably curbed these behaviors and induced mitochondrial depolarization, along with ROS production, culminating in apoptosis. Moreover, the absence of TEFM substantially influenced the expression of mitochondrial transcripts and respiratory chain complexes. Results from nude mouse xenograft tumors further validated that inhibiting TEFM expression markedly hindered tumor growth.

**Conclusion:**

TEFM promotes LUAD malignant progression through the EMT pathway and determines apoptosis by affecting the expression of mitochondrial transcripts and respiratory chain complexes, providing a new therapeutic direction for LUAD-targeted therapy.

**Graphical Abstract:**

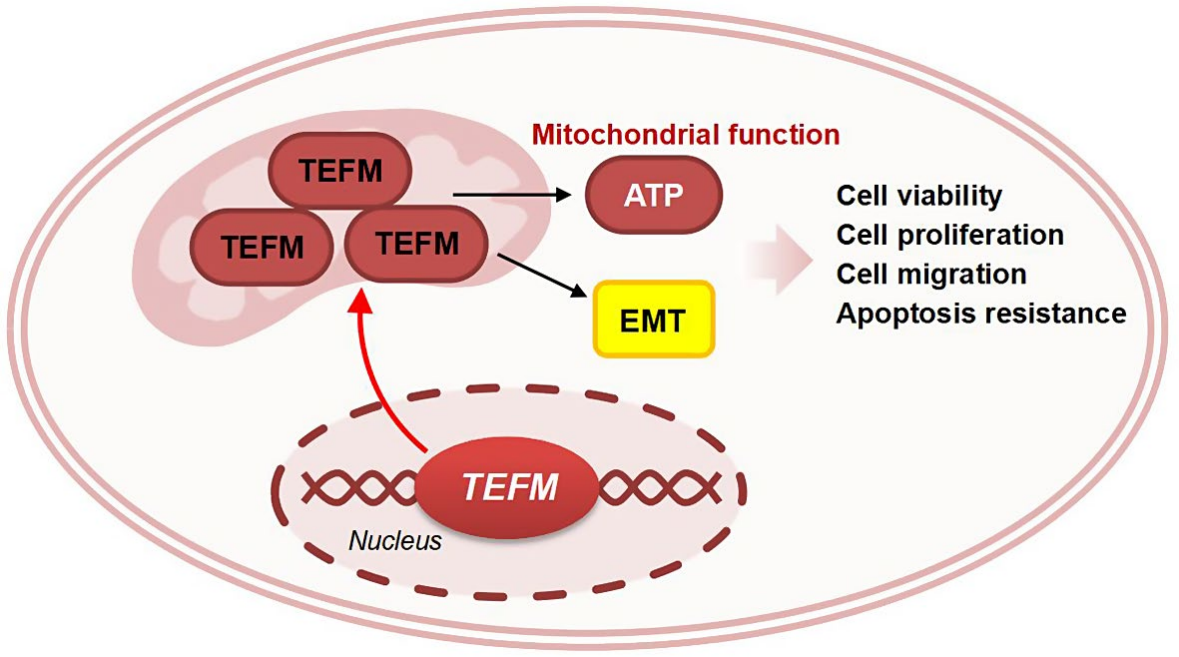

**Supplementary Information:**

The online version contains supplementary material available at 10.1186/s12967-024-05483-2.

## Introduction

Although screening techniques and treatments are continually advancing, lung cancer remains one of the deadliest cancers globally [1]. Among all types of lung cancer, non-small cell lung cancer (NSCLC) constitutes approximately 85% of cases [2]. Lung adenocarcinoma (LUAD) accounts for the vast majority of NSCLC.While molecularly targeted therapies and immunotherapy have significantly enhanced the prognosis of LUAD patients, not all individuals with LUAD possess suitable molecular targets [3–5]. Hence, there is an urgent need to explore new potential molecules for predicting LUAD prognosis and to develop effective targeted therapeutic strategies against it. In recent years, mitochondria have emerged as a hotspot in human cancer research [6]. They participate in most metabolic activities of mammalian cells and play a central role in maintaining redox homeostasis and regulating apoptosis [7]. Recent evidence suggests that active mitochondrial metabolism is essential for tumor growth, and targeting mitochondrial function represents a novel approach to cancer therapy [8].

Essential components of transcription in the human mitochondrial genome have been identified, including mitochondrial RNA polymerase (POLRMT), mitochondrial transcription factor A (TFAM), and B2 (TFB2M). These proteins collectively participate in initiating mitochondrial transcription [9, 10]. The process of elongation is primarily regulated by the mitochondrial transcription elongation factor (TEFM) [11–16]. Deletion of TEFM affects the processing of mitochondrial RNA in mammals [17], further impacting the normal functioning of multiple biological processes. Although abnormal mitochondrial transcription has been associated with various human diseases such as diabetes, congenital myasthenia gravis, and cardiovascular disease, its role in cancer remains poorly understood [18, 19]. Wan et al. reported elevated TEFM expression in hepatocellular carcinoma, which promotes growth and metastasis [20]. However, TEFM’s role in other tumors remains unknown.

The present study unveils abnormally high TEFM expression in LUAD (Lung Adenocarcinoma), closely linked with poor patient prognosis. It elucidates the potential mechanism by which TEFM expression functions in LUAD.

## Materials and methods

### Cells

BEAS-2B, A549, H1299, and H1650 cell lines were obtained from the laboratory of Thoracic Surgery, the First Affiliated Hospital of Soochow University, while primary human NSCLC cells were provided by Dr. Xu [21] at Soochow University. Previously published protocols were followed for culturing these cells [21–23]. Ethical approval for the use of primary human NSCLC cells was obtained from the Ethics Committee of the First Affiliated Hospital of Soochow University, adhering to the principles of the Declaration of Helsinki.

### Reagents

The TEFM antibody (PA5-97096) was procured from Thermo-Fisher Invitrogen (Shanghai, China), while puromycin, fetal bovine serum, antibiotics, and other cell culture reagents were sourced from Sigma (St. Louis, Mo). Cox1(PA5-95171), Cytb (PA5-100740), NDUFB8 (459,210) and Immunofluorescent Staining Related Antibody(Mito Tracker Red (A6643),DAPI (62,248)) were also purchased from Thermo-Fisher Invitrogen (Shanghai, China).

All fluorescent dye reagents were supplied by Thermo-Fisher Invitrogen (Shanghai, China), and other pertinent antibodies (E-Cadherin (3195S0, N-Cadherin (13,116 S), Vimentin (5741 S)) were obtained from Cell Signaling Technologies (Beverly, MA).

### Clinical specimen

LUAD tissues and their corresponding adjacent normal tissues were collected from 21 patients with primary LUAD (provided by Dr. Xu from the First Affiliated Hospital of Soochow University [21, 23]. The paired adjacent normal lung specimens were 1.2 cm from the edge of the tumour. Fresh tissue specimens were preserved in liquid nitrogen immediately after surgery. Written informed consent was obtained from all patients. The experimental protocol involving human tissues was approved by the Ethics Committee of the First Affiliated Hospital of Soochow University.

### TEFM ShRNA or overexpression

Lentiviral constructs containing shRNAs targeting non-overlapping sequences of TEFM (shTEFM-1, shTEFM-2) and lentiviral constructs encoding full-length TEFM cDNAs (OE-TEFM) were procured from Genechem (Shanghai, China). The specific knockdown sequences for TEFM were 5-GTGAAGCAGTTTCTCTTCGAT-3 (shTEFM-1) and 5-GAGCATGAATCGAAATGCAGT-3 (shTEFM-2).

### Cell Counting Kit-8 (CCK-8)

200 µL of cells, containing 2 × 10^3, was slowly added dropwise to a 96-well plate. After incubation for the indicated duration, 20 µL of CCK-8 reagent was added to each well, and the absorbance was measured using an enzyme marker after two hours of further incubation.

### Cell migration

We utilized Transwell chambers obtained from (Corning, NY) for our experiments. These chambers were positioned in 24-well plates, and 500 µL of cell culture medium containing 10% fetal bovine serum was added to the lower chamber. Subsequently, cells resuspended in pure medium (300 µL containing 2 × 10^4 cell number) were slowly added to the chambers. After incubation for the specified duration, the cells were fixed and stained using crystal violet dye. The chambers were then washed three times in PBS, and the inside of the chambers were wiped with cotton swabs. After the chambers were dried, they were photographed under an inverted microscope, and the number of cells in one field of view was counted randomly.

### Xenograft

Experiments were conducted using 5-6-week-old nude mice obtained from GemPharmatech Co. Cells in the logarithmic phase of growth were taken, digested down and then washed twice with pre-cooled PBS to remove any residual serum from the cells. Two million cells were resuspended in serum-free medium and mixed with matrix gel to prepare a 150 µL suspension. Cell suspensions should be placed on ice after preparation to reduce cell metabolic activity (try to inoculate mice subcutaneously within half an hour). This suspension was then injected into the right axilla of nude mice. The mice were divided into two groups (Cas9C and koTEFM) based on the injected cells, and their body weights and subcutaneous graft tumor volumes were recorded every 5 days. On day 30, the mice were euthanized, and the graft tumors were excised and photographed. All animal experiments were approved by the Animal Ethics Review Committee of Soochow University.

### Other assays

The comprehensive procedures for other experiments, including western blotting, colony formation assays, qRT-PCR, single-stranded DNA (ssDNA), EdU, TUNEL, JC-1, flow cytometry, ROS staining, have been thoroughly described in previously published studies [21–23].

### Bioinformatics analysis

All bioinformatics analyses were completed using R studio 4.2.1. Gene expression data for all lung adenocarcinoma patient tumour tissues and normal tissues were obtained from the TCGA public database (https://portal.gdc.cancer.gov/).

### Statistical analyses

The data presented in this article adhere to a normal distribution and are expressed as mean ± standard deviation (SD). Statistical t-tests and data analyses were performed utilizing GraphPad Prism 6 software. All experimental procedures were replicated thrice.

**Table 1:** Sequences utilized in this study

Gene name	qRT-PCR primer forward (5’-3’)	qRT-PCR primer reverse (5’-3’)
TEFM	ATGAGCGGGTCTGTCCTCTT	AGTACAGGGATGACCTCGACG
Cytb	ATCACTCGAGACGTAAATTATGGCT	TGAACTAGGTCTGTCCCAATGTATG
Cox1	TCTCAGGCTACACCCTAGACCA	ATCGGGGTAGTCCGAGTAACGT
NDUFB8	TACAACAGGAACCGTGTGGA	CTGGTTCTTTGGAGGGATCA
β-actin	TCGCCTTTGCGATCCG	ATGATCTGGGTCATCTTCTCG
sgRNA	Target DNA sequence	PAM sequence
TEFM sgRNA	GAAAACCGGTTCCTGAGAAA	CCG

## Results

### TEFM is highly expressed in human LUAD and is associated with poor patient prognosis

**Fig. 1:**
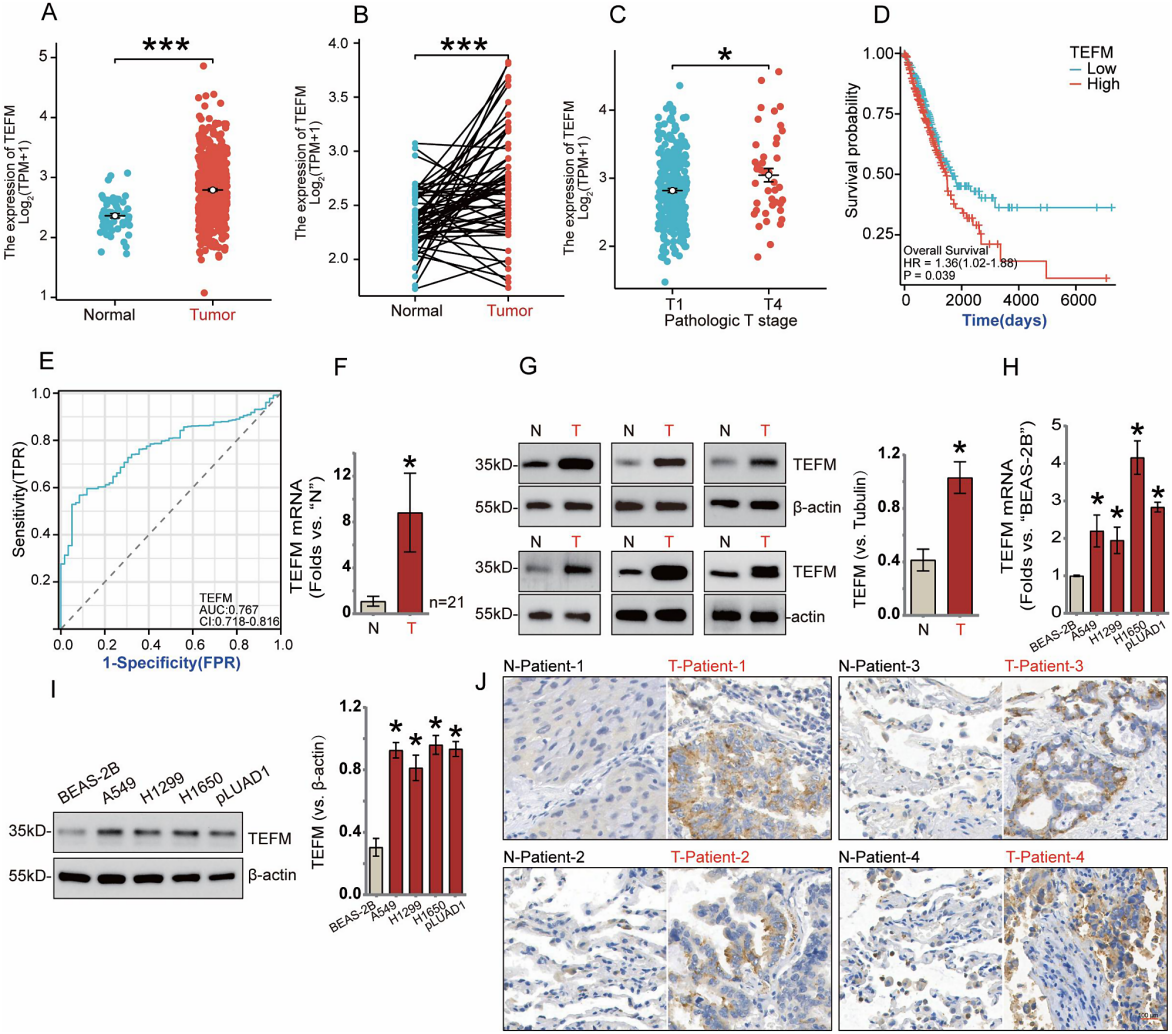
TEFM is highly expressed in human LUAD and is associated with poor patient prognosis. The relative expression of mRNA for TEFM in unpaired (**A**) and paired samples of LUAD tissue and normal tissue was analyzed using the TCGA database. The relative expression of mRNA for TEFM in T1 and T4 stages of NSCLC pathological T-stages is also shown (**C**). Subsequently, survival curves of the patients were plotted according to the high and low expression of TEFM in LUAD (**D**), and ROC curves were plotted to assess the accuracy of the modified survival curve model (**E**). The mRNA expression of TEFM in tumour tissues (“T”) and paired surrounding normal lung epithelial tissues (“N”) from 21 patients with LUAD is shown (**F**), along with the protein expression and quantification of TEFM in 6 representative pairs of patients (**G**). Furthermore, the mRNA (**H**) and protein expression (**I**) and quantification of TEFM in established human normal lung epithelial cell line (BEAS-2B) and human NSCLC cell lines (A549, H1299, H1650) as well as primary human LUAD cells (pLUAD1) are depicted. Finally, immunohistochemical staining plots of TEFM in tumour tissues and paired normal tissues from 4 typical NSCLC patients are shown (**J**). Data were presented as mean ± standard deviation (SD, *n* = 3). *P < 0.05 versus “Normal”/“N”. The experiments were repeated thrice. Scale bar = 20 μm. β-actin was used as an internal reference

We initially analyzed the mRNA levels of TEFM using The Cancer Gene Atlas (TCGA) database and observed significantly elevated expression of TEFM in LUAD tissues compared to normal lung tissues in both unpaired (Fig. 1A) and paired samples (Fig. 1B). Moreover, TEFM expression was found to correlate with the clinicopathological T-stage of NSCLC, with higher expression observed in patients with T4 stage compared to those with T1 stage (Fig. 1C). Additionally, patients exhibiting high TEFM expression had significantly shorter survival times compared to those with low TEFM expression (Fig. 1D), suggesting a potential association between TEFM and tumor progression, which was further supported by survival model tests (Fig. 1E).

To validate these bioinformatics findings, we performed qRT-PCR to assess TEFM mRNA expression in tumor tissues and paired paracancerous tissues obtained from 21 LUAD patients from the Department of Thoracic Surgery of the First Affiliated Hospital of Soochow University. Consistently, TEFM expression was significantly higher in cancer tissues compared to normal lung tissues (Fig. 1F). Western blot analysis further confirmed the upregulation of TEFM expression in representative NSCLC patients (Fig. 1G).

Furthermore, we investigated TEFM expression in NSCLC cells, revealing significantly higher mRNA (Fig. 1H) and protein (Fig. 1I) levels in established A549, H1299, H1650, as well as patient-derived primary human NSCLC cells (pLUAD1) compared to lung epithelial tissue BEAS-2B cells.

Finally, representative immunohistochemical staining images from 4 patients (Patient-1, Patient-2, Patient-3, Patient-4) further supported the higher expression of TEFM protein in LUAD tissues relative to paired normal lung epithelial tissues (Fig. 1J). Collectively, these findings indicate an upregulation of TEFM expression in human LUAD.

### Overexpression of TEFM promotes proliferation and migration of human LUAD cells

**Fig. 2:**
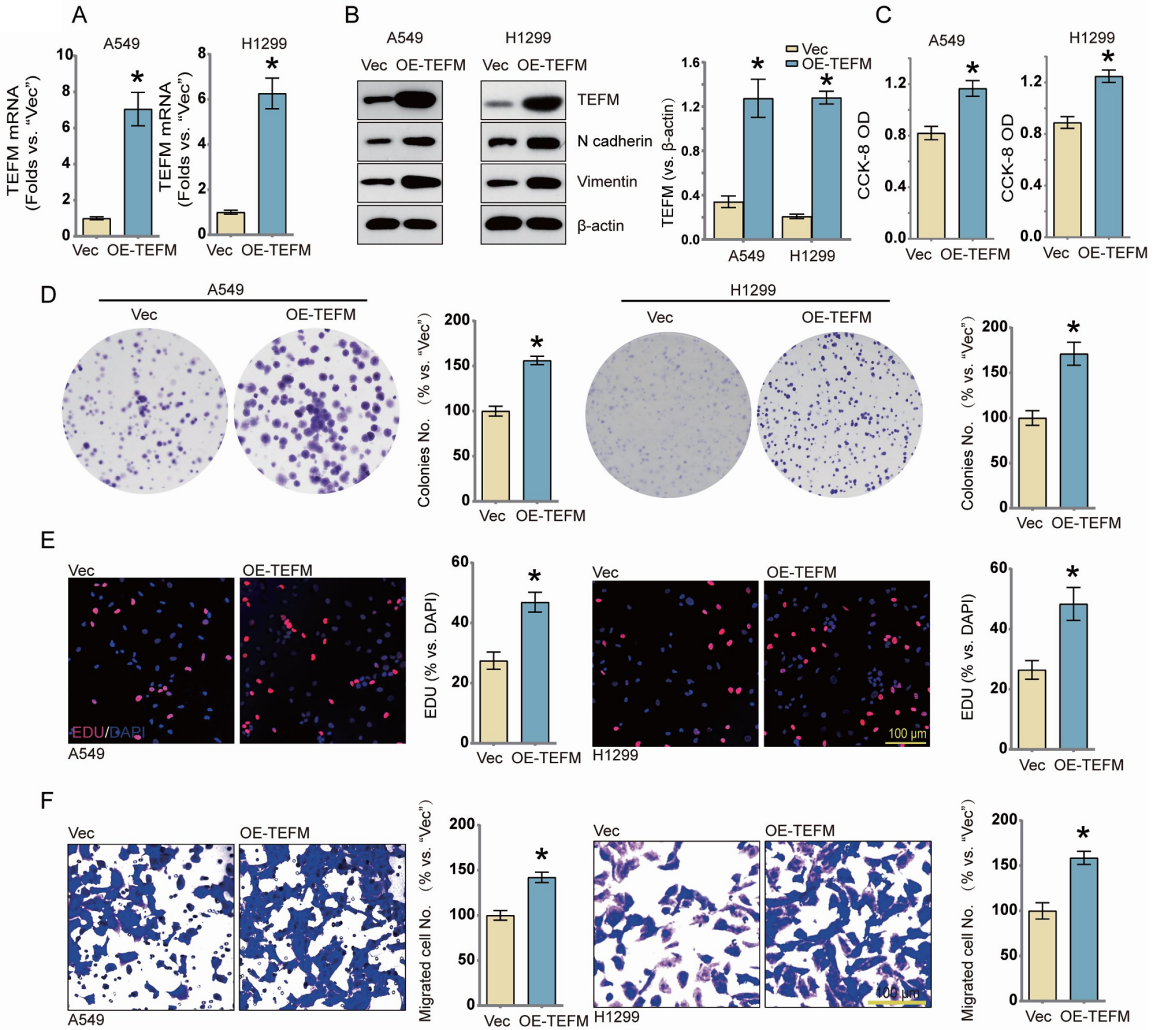
Overexpression of TEFM promotes proliferation and migration of human LUAD cells. Groups of TEFM overexpressing (OE-TEFM) A549 and H1299 cells and empty control cells were established (Vec). The mRNA and protein expression levels of TEFM were detected (**A**, **B**) as well as the expression of EMT pathway-related markers (**B**). Cells were cultured for the indicated times and then tested for cell CCK-8 OD values, colony formation, cell proliferation (nuclear EdU staining), and migration ability (Transwell), and the results were quantified and analyzed. Data were presented as mean ± standard deviation (SD, *n* = 3). *P < 0.05 versus “Vec”. The experiments were repeated thrice. Scale bar = 100 μm. β-actin was used as an internal reference

To investigate the potential effects of TEFM on LUAD cells, we generated lentiviruses encoding TEFM cDNA (OE-TEFM) and control lentiviruses (Vec). Following transfection of A549 and H1299 cells with these constructs, we screened and established stably transfected cell lines using puromycin selection. Subsequent verification of TEFM expression in stably transfected cells by qRT-PCR (Fig. 2A) and western blotting (Fig. 2B) confirmed significant upregulation of (OE-TEFM) expression in TEFM-overexpressing LUAD cells.

We then conducted functional experiments to assess alterations in malignant behavior. The OD values of CCK8 in A549 and H1299 cells overexpressing TEFM were markedly higher compared to the control group (Fig. 2C), indicative of increased cell viability. Moreover, colony formation assays revealed effective inhibition of colony formation in the TEFM overexpression group (Fig. 2D). Additionally, the percentage of EdU-positive nuclei was significantly elevated (Fig. 2E), supporting the notion that TEFM overexpression effectively promotes the proliferation of NSCLC cells.

Furthermore, the migratory ability of LUAD cells overexpressing TEFM was notably enhanced, as demonstrated by Transwell assay (Fig. 2F). This observation was further corroborated by the upregulation of N-cadherin and Vimentin protein expression detected by western blotting (Fig. 2B).

### TEFM deficiency inhibits proliferation and migration of human LUAD cells

**Fig. 3:**
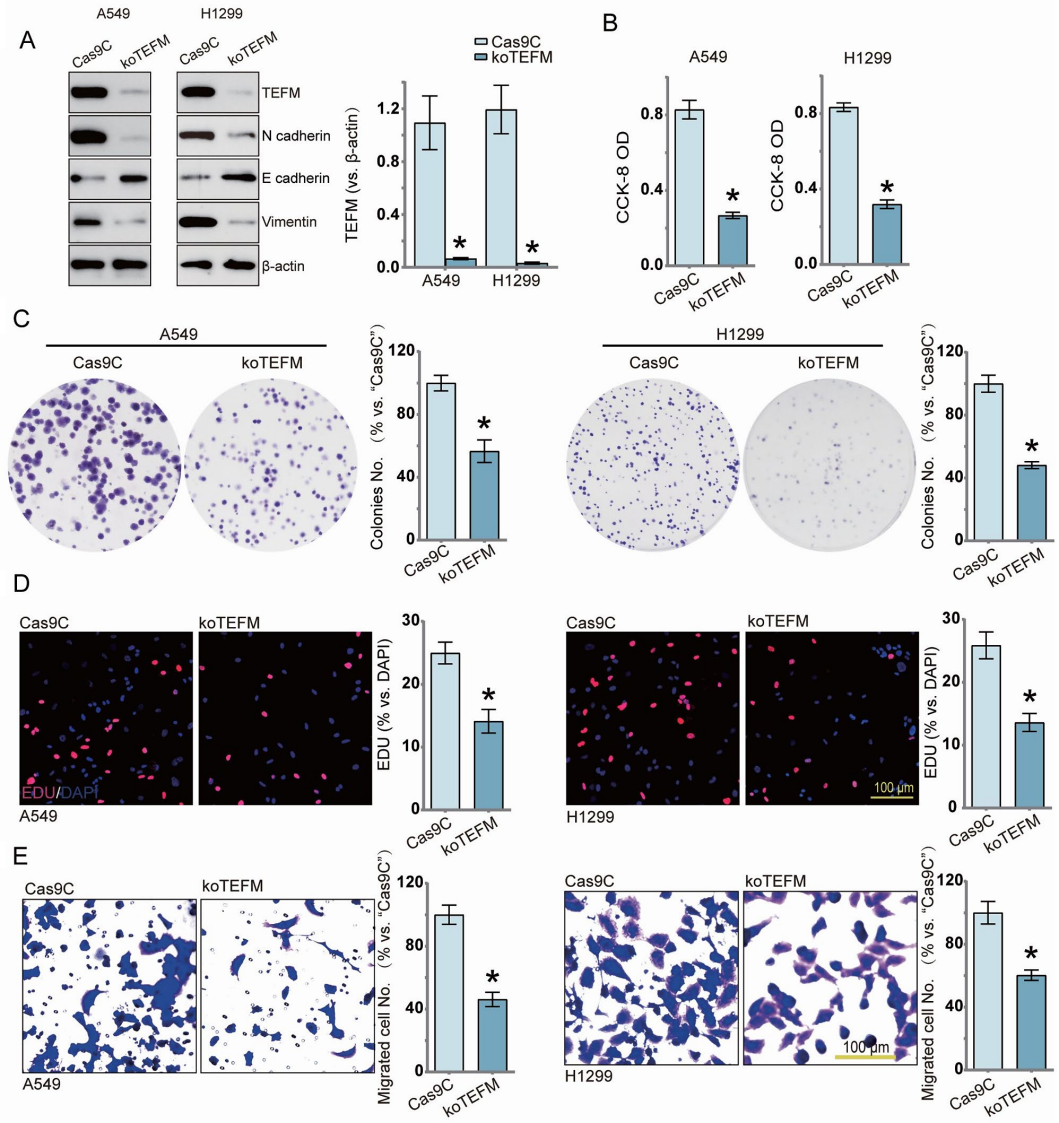
TEFM deficiency inhibits proliferation and migration of human LUAD cells. Stably transfected cell lines were established using a TEFM sgRNA lentiviral construct (koTEFM) and a control airborne lentivirus (Cas9C). Protein expression of TEFM and EMT-related markers is shown (**A**). After a period of culture, cell viability (CCK-8 OD) (**B**), colony formation (**C**), proliferation (percentage of EdU-positive nuclei) (**D**), and cell migration ability (**E**) were tested and quantified for the above results. Data were presented as mean ± standard deviation (SD, *n* = 3). *P < 0.05 versus “Cas9C”. The experiments were repeated thrice. Scale bar = 100 μm. β-actin was used as an internal reference

To further elucidate the effect of TEFM deletion on NSCLC cell function, we constructed two lentiviruses (shTEFM-1, shTEFM-2) and a negative control lentivirus (shC) carrying knockdown sequences of the TEFM gene. And we employed a CRISPR/Cas9 gene editing strategy established TEFM knockout sequences (koTEFM) and control negative sequences (Cas9C). A549 and H1299 cells were transfected with CRISPR/Cas9-TEFM-KO lentiviral constructs and shRNA lentiviral constructs, and expression of TEFM and mesenchymal transition marker proteins was verified in stable knockout and knockdown cell lines (Fig. 3A,S1A). As shown, CCK-8 assay, cell colony formation, Edu staining and Transwell assay demonstrated that CRISPR/Cas9-mediated TEFM-KO as well as shRNA-mediated shTEFM inhibited LUAD cell activity (Fig. 3B, S1B), colony formation (Fig. 3C, S1C), proliferation (Fig. 3D, S1D), and migratory ability (Fig. 3E, S1E), respectively.

### TEFM deficiency causes disruption of mitochondrial function and activation of apoptosis

**Fig. 4:**
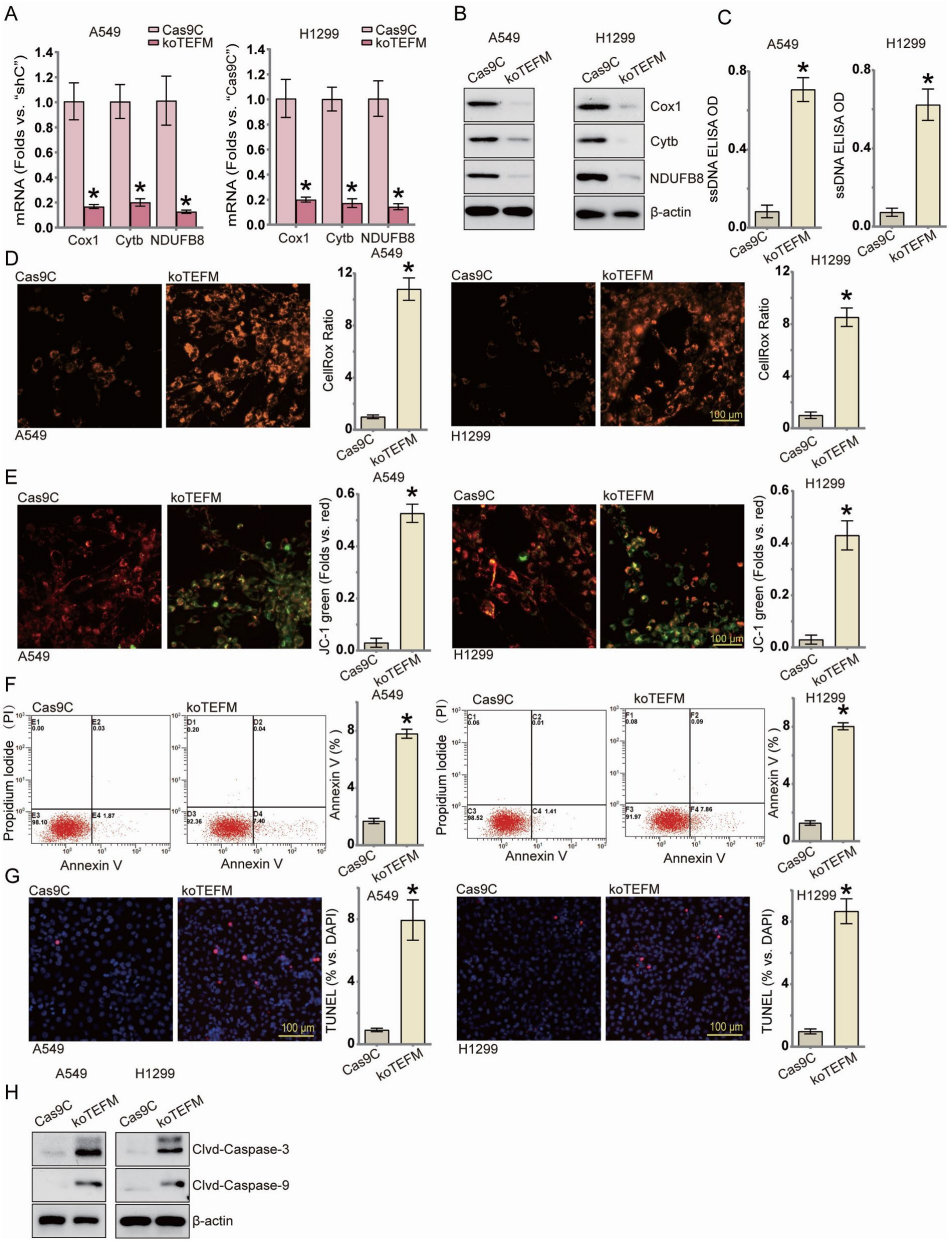
TEFM deficiency causes disruption of mitochondrial function and activation of apoptosis.A549 and H1299 cells transfected with TEFM sgRNA lentivirus (koTEFM) and control null lentivirus (Cas9C) were cultured. Subsequently, mRNA (**A**) and protein expression levels (**B**) of the mitochondrial transcript Cytb and the mitochondrial respiratory chain complexes Cox1 and NDUFB8 were examined in cells of the TEFM knockout group. Single-stranded DNA (ssDNA) content was assayed (**C**) and cellular ROS levels were measured by CellRox (**D**). The extent of mitochondrial depolarisation was shown by detecting the intensity of JC-1 green monomer (**E**). The level of apoptosis in the relevant cells was detected by flow cytometry (**F**) and TUNEL-positive nuclear ratios (**G**) as well as caspase-3 and caspase-9 cleavage protein expression levels (**H**), and all the results are presented quantitatively. Data were presented as mean ± standard deviation (SD, *n* = 3). *P < 0.05 versus “Vec”. The experiments were repeated thrice. Scale bar = 100 μm. β-actin was used as an internal reference

We hypothesized that TEFM deficiency, being integral to mitochondrial transcription, might reduce mitochondrial transcripts. Using qRT-PCR and Western blotting, we evaluated expression levels of mitochondrial respiratory chain complex subunits. Our findings demonstrated a significant decrease in both mRNA content (Fig. 4A, S2A) and protein levels (Fig. 4B, S2B) of Cox1, Cytb, and NDUFB8 subunits in TEFM-deficient A549 and H1299 cells. Furthermore, TEFM deletion led to heightened levels of single-stranded DNA (Fig. 4C, S2C).

Subsequent analysis revealed a notable increase in intracellular reactive oxygen species levels in TEFM-deficient cells compared to controls (Fig. 4D, S2D), accompanied by induced mitochondrial depolarization and reduced mitochondrial membrane potential levels (Fig. 4E, S2E). Concurrently, we observed a rise in the proportion of TUNEL-positive nuclei (Fig. 4G, S2G) and Annexin V-positive staining (Fig. 4F, S2F), indicative of apoptosis. Moreover, assessment of cysteine asparaginase activities in TEFM knockout and knockdown A549 and H1299 (Fig. 4H, S2H) cells via Western blotting revealed a significant increase in the cleavage products of caspase-3 and caspase-9. These cumulative findings affirm the activation of apoptosis consequent to TEFM deletion.

### Apoptosis due to TEFM deficiency is caused by elevated reactive oxygen species

**Fig. 5:**
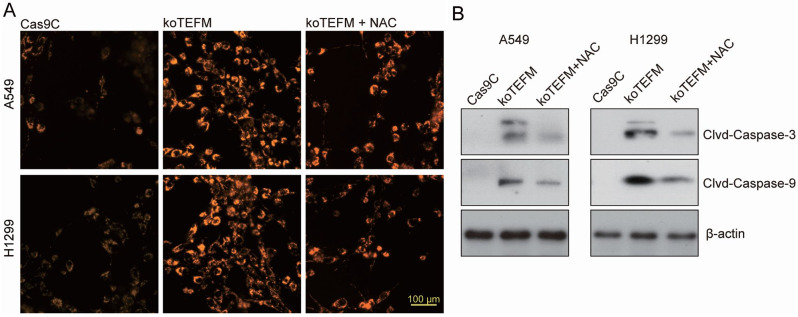
Apoptosis due to TEFM deficiency is caused by elevated reactive oxygen species. A549 and H1299 cells transfected with TEFM sgRNA lentivirus (koTEFM) and control null lentivirus (Cas9C) were cultured. Cellular reactive oxygen species levels were detected after 24 h of treatment with the mitochondrial ROS scavenger N-acetylcysteine (NAC, 5 mM) (**A**), and the expression levels of caspase-3 and caspase-9 shear body proteins were detected by western blotting (**B**). Data were presented as mean ± standard deviation (SD, *n* = 3). *P < 0.05 versus “Cas9C”. The experiments were repeated thrice. Scale bar = 100 μm. β-actin was used as an internal reference

To ascertain if apoptosis triggered by TEFM deletion stems from heightened intracellular reactive oxygen species (ROS), we investigated cellular ROS levels following treatment of TEFM knockout cells with the mitochondrial ROS scavenger N-acetylcysteine (NAC, 5 mM) for 24 h [24]. Remarkably, results revealed a significant reduction in ROS levels in cells treated with NAC compared to TEFM knockout cells alone (Fig.  5)A.

Subsequently, we scrutinized the expression levels of apoptosis-related proteins via Western blotting. Strikingly, the cleavage products of caspase-3 and caspase-9 in TEFM knockout cells exhibited significant reduction post NAC treatment. These findings robustly substantiate that TEFM deletion instigates apoptosis through ROS elevation (Fig. 5B).

### TEFM KO inhibits the growth of human LUAD cells in nude mice in vivo

**Fig. 6:**
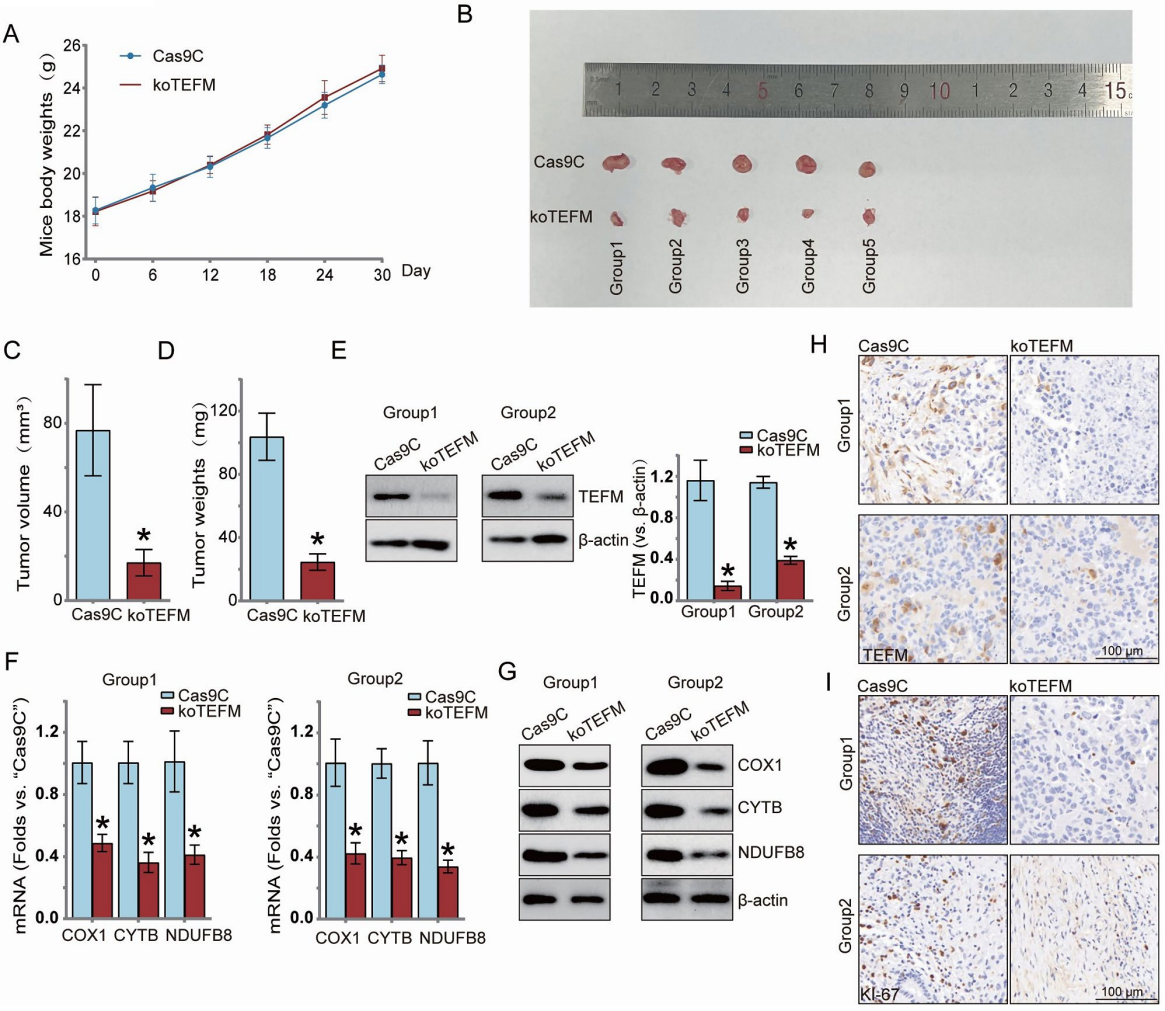
TEFM KO inhibits the growth of human LUAD cells in nude mice in vivo. Ten nude mice were randomly divided into 2 groups (5 mice in each group) and injected (day 0) using TEFM knockout (koTEFM) or blank control (Cas9C) H1299 cells, followed by measuring the weights of the mice at 6-day intervals (**A**), and removing the xenograft tumours for photographing on day 30 (**B**), and recording the tumour volume (**C**) and weight (**D**). Mouse tumours were divided into five groups, and two groups were selected for quantitative analysis of the results using western blotting and qRT-PCR to detect the levels of TEFM (**E**) as well as mRNA (**F**) and protein expression (**G**) of mitochondria-associated transcripts and respiratory chain complexes. Immunohistochemical staining was performed on the xenograft tumours of the two groups of nude mice to detect the expression of TEFM (**H**) and KI-67 (**I**) in the tumour tissues, with brown representing positive staining.Data were presented as mean ± standard deviation (SD, *n* = 3). *P < 0.05 versus “Cas9C”. The experiments were repeated thrice. β-actin was used as an internal reference

To investigate the effect of TEFM on the growth of LUAD cells in vivo, we injected TEFM knockout (KOTEFM) and blank control (Cas9C) H1299 cells at 2 × 10^6 into the right axilla of nude mice, respectively, to establish the experimental group and the control group (“0 day”). At 6-day intervals, the body weights of the two groups of mice were recorded, revealing no significant difference in body weight between the groups (Fig. 6A). On the thirtieth day, the mice were euthanized, and the transplanted tumors were removed and photographed for documentation (Fig. 6B), followed by weighing of the tumors (Fig. 6C) and calculation of tumor volume (Fig. 6D). Results indicated that compared to the control group, the transplanted tumors in the TEFM knockout group were significantly lighter in weight and smaller in volume. The graft tumors were further divided into 5 groups (Group1-5) based on experimental treatments, with Group1 and Group2 selected as representatives for detection of TEFM protein expression using western blotting. This analysis revealed significant downregulation of TEFM protein expression in koTEFM-injected xenografts (Fig. 6E). Additionally, mRNA expression (Fig. 6F) and protein expression (Fig. 6G) levels of the mitochondrial transcript Cytb and the mitochondrial respiratory chain complex Cox1, NDUFB8, were significantly reduced in xenografts of the TEFM knockout group. Finally, we performed immunohistochemical staining of the two groups of xenografts, which showed that the expression of TEFM was significantly downregulated in the TEFM knockout group (Fig. 6H), and KI-67 staining showed that koTEFM was able to considerably reduce the proliferative viability of cells (Fig. 6I).Thus, consistent with in vitro studies, TEFM knockout led to reduced expression of mitochondrial transcripts and mitochondrial respiratory chain complexes in LUAD xenografts.

To further validate the results of the in vivo experiments, we inoculated 12 nude mice with H1299 cells overexpressing TEFM (OE-TEFM) and their respective control group of H1299 cells (Vec) using the same method. The weight of the nude mice was measured every 5 days, and on the 15th day, the mice were euthanized (Fig. S3A), and the transplanted tumors were excised and photographed for documentation (Fig. S3B). The results showed that the tumors overexpressing TEFM exhibited significantly greater weight (Fig. S3C) and volume (Fig. S3D) compared to the control group. These findings further corroborate the role of TEFM in promoting tumor cell growth in vivo.

## Discussion

Mitochondrial oxidative phosphorylation (OXPHOS) is is a major pathway for ATP production in cells, and it has recently been shown that OXPHOS plays a key role in providing the bioenergetic and macromolecular anabolic requirements of cancer cells [25–27]. It is up-regulated in a variety of cancers to meet the energy requirements for tumourigenesis and progression [28, 29], and increased OXPHOS dependence is often a marker of tumour cell resistance to chemotherapy and targeted therapy [30, 31]. Therefore, targeting OXPHOS proteins and inhibiting mitochondrial respiration may provide a novel option to inhibit the malignant progression of tumour cells [32–34].

Previous studies have shown that TEFM is essential for mitochondrial biosynthesis [17], and Wan et al. showed that TEFM deficiency inhibited metastasis of hepatocellular carcinoma [20].

The results of the present study support that TEFM may be a new and important therapeutic target for LUAD, and we found that TEFM expression was up-regulated in LUAD tissues and cells, and that high TEFM expression was strongly associated with poor patient prognosis. Ectopic overexpression of TEFM by lentiviral constructs enhanced the proliferation and motility of LUAD cells, in which the EMT pathway played a key role. In contrast, TEFM silencing induced by shRNA or TEFM knockout induced by CRISPR/Cas9 effectively suppressed cell proliferation, viability, and migration ability, and induced cysteinyl asparagin-apoptosis activation. Importantly, we found that TEFM deletion caused mitochondrial DNA transcriptional repression and led to a significant reduction in mRNA expression of mitochondrial respiratory chain complex subunits (Cox1, CYTB, NDUFB8), mitochondrial depolarisation and reactive oxygen species generation, and the above results suggest that TEFM is a key molecule indispensable for mitochondrial synthesis.

Increasing evidence suggests that targeting OXPHOS in tumour cells may lead to severe mitochondrial dysfunction and energetic crisis, which in turn inhibits cancer cell progression [35, 36].TEFM acts as a mitochondrial transcriptional elongation factor, which is critical for mtDNA transcription, OXPHOS and mitochondrial biological processes. Here, we found that TEFM promotes the migratory behaviour of tumour cells through EMT, and its high expression meets the high metabolic state of tumour cells by maintaining high mitochondrial function and enhances the proliferation of tumour cells. Therefore, we propose that inducing mitochondrial dysfunction and energetic crisis in tumour cells by targeting TEFM may be a novel strategy for the treatment of LUAD.

Indeed, discussions surrounding therapeutic strategies and targeting methods for TEFM necessitate further exploration. TEFM plays a crucial role in the normal growth of all cells, and its inhibition can lead to various toxicities (PMID: 31,036,713),suggesting potential serious side effects if directly targeting the TEFM gene for editing. Thus, exploring transcription factors regulating TEFM expression or targeting the proteasome responsible for TEFM degradation presents a novel avenue. By indirectly modulating TEFM expression, we could potentially mitigate treatment-related side effects.

Furthermore, advancements in nanotechnology and biotechnology have propelled research on material delivery for targeted tumor therapy significantly. Leveraging nanodelivery systems like liposomes, polymer nanoparticles, and other technological means holds promise in effectively targeting the TEFM gene within tumor cells. Such approaches could enhance targeted delivery efficiency while substantially minimizing associated side effects.

Our study delineates TEFM as a promising therapeutic target in LUAD cells. However, the development of therapeutic tools for TEFM is imperative to translate this potential into clinical applications.

## Conclusion

Significant strides have been made in the treatment of LUAD in recent years, with active exploration of targeted therapies. However, the emergence of drug resistance and late metastasis underscores the urgent need for new interventions [4, 5]. Our study suggests that targeting TEFM could represent a novel therapeutic approach to curb the malignant progression of LUAD cells.

### Electronic supplementary material

Below is the link to the electronic supplementary material.

Supplementary Material 1

Supplementary Material 2

Supplementary Material 3

## Data Availability

All data are available in the main text or the supplementary materials. Further inquiries can be addressed to the corresponding author.

## Abbreviations

NSCLC: Nonsmall cell lung cancer
LUAD: Lung Adenocarcinoma
TEFM: Mitochondrial transcription elongation factor
EMT: Epithelial-mesenchymal transition
POLRMT: Mitochondrial RNA polymerase
TFAM: Mitochondrial transcription factor A
TFB2M: Mitochondrial transcription factor B2
ssDNA: Single-stranded DNA
OXPHOS: Oxidative phosphorylation
mtDNA: mitochondrial DNA
NAC: N-acetylcysteine

## References

[1] Nasim F, Sabath BF, Eapen GA. Lung Cancer Med Clin North Am. 2019;103(3):463–73. 30955514 10.1016/j.mcna.2018.12.006

[2] Gridelli C, et al. Non-small-cell lung cancer. Nat Rev Dis Primers. 2015;1:15009. 27188576 10.1038/nrdp.2015.9

[3] Shi Y, Shin DS. Dysregulation of SWI/SNF chromatin remodelers in NSCLC: its influence on Cancer therapies including Immunotherapy. Biomolecules, 2023. 13(6). 10.3390/biom13060984 PMC10296130 37371564

[4] Srivastava S, et al. Chemokines and NSCLC: emerging role in prognosis, heterogeneity, and therapeutics. Semin Cancer Biol. 2022;86(Pt 2):233–46. 35787939 10.1016/j.semcancer.2022.06.010

[5] Alexander M, Kim SY, Cheng H. Update 2020: management of Non-small Cell Lung Cancer. Lung. 2020;198(6):897–907. 33175991 10.1007/s00408-020-00407-5 PMC7656891

[6] Roth KG, et al. The Mitochondrion as an emerging therapeutic target in Cancer. Trends Mol Med. 2020;26(1):119–34. 31327706 10.1016/j.molmed.2019.06.009 PMC6938552

[7] Zhang H, et al. Systematic investigation of mitochondrial transfer between cancer cells and T cells at single-cell resolution. Cancer Cell. 2023;41(10):1788–e180210. 37816332 10.1016/j.ccell.2023.09.003 PMC10568073

[8] Vasan K, Werner M, Chandel NS. Mitochondrial metabolism as a target for Cancer Therapy. Cell Metab. 2020;32(3):341–52. 32668195 10.1016/j.cmet.2020.06.019 PMC7483781

[9] Minczuk M, et al. TEFM (c17orf42) is necessary for transcription of human mtDNA. Nucleic Acids Res. 2011;39(10):4284–99. 21278163 10.1093/nar/gkq1224 PMC3105396

[10] Hillen HS, et al. Structural basis of mitochondrial transcription initiation. Cell. 2017;171(5):1072–e108110. 29149603 10.1016/j.cell.2017.10.036 PMC6590061

[11] Posse V, et al. TEFM is a potent stimulator of mitochondrial transcription elongation in vitro. Nucleic Acids Res. 2015;43(5):2615–24. 25690892 10.1093/nar/gkv105 PMC4357710

[12] Hillen HS, et al. Mechanism of transcription anti-termination in human mitochondria. Cell. 2017;171(5):1082–e109313. 29033127 10.1016/j.cell.2017.09.035 PMC5798601

[13] Barshad G, et al. Mitochondrial DNA transcription and its regulation: an evolutionary perspective. Trends Genet. 2018;34(9):682–92. 29945721 10.1016/j.tig.2018.05.009

[14] Hillen HS, Temiakov D, Cramer P. Structural basis of mitochondrial transcription. Nat Struct Mol Biol. 2018;25(9):754–65. 30190598 10.1038/s41594-018-0122-9 PMC6583890

[15] Bouda E, Stapon A, Garcia-Diaz M. Mechanisms of mammalian mitochondrial transcription. Protein Sci. 2019;28(9):1594–605. 31309618 10.1002/pro.3688 PMC6699104

[16] Agaronyan K, et al. Mitochondrial biology. Replication-transcription switch in human mitochondria. Science. 2015;347(6221):548–51. 25635099 10.1126/science.aaa0986 PMC4677687

[17] Jiang S et al. TEFM regulates both transcription elongation and RNA processing in mitochondria. EMBO Rep, 2019. 20(6). 10.15252/embr.201948101 PMC6549021 31036713

[18] Lei T, et al. Mitochondria transcription and cancer. Cell Death Discov. 2024;10(1):168. 38589371 10.1038/s41420-024-01926-3 PMC11001877

[19] O’Connor K et al. Mitochondrial mutations can alter neuromuscular transmission in congenital myasthenic syndrome and mitochondrial disease. Int J Mol Sci, 2023. 24(10). 10.3390/ijms24108505 PMC10218558 37239850

[20] Wan L, et al. Elevated TEFM expression promotes growth and metastasis through activation of ROS/ERK signaling in hepatocellular carcinoma. Cell Death Dis. 2021;12(4):325. 33771980 10.1038/s41419-021-03618-7 PMC7997956

[21] Chen TF, et al. HBO1 induces histone acetylation and is important for non-small cell lung cancer cell growth. Int J Biol Sci. 2022;18(8):3313–23. 35637972 10.7150/ijbs.72526 PMC9134900

[22] Zhou T, et al. The requirement of mitochondrial RNA polymerase for non-small cell lung cancer cell growth. Cell Death Dis. 2021;12(8):751. 34326320 10.1038/s41419-021-04039-2 PMC8322058

[23] Cui Z, et al. The sodium/myo-inositol co-transporter SLC5A3 promotes non-small cell lung cancer cell growth. Cell Death Dis. 2022;13(6):569. 35760803 10.1038/s41419-022-05017-y PMC9237060

[24] Roca MS et al. HDAC class I inhibitor domatinostat sensitizes pancreatic cancer to Ch emotherapy by targeting cancer stem cell compartment via FOXM1 modulat ion. J Experimental Clin cancer Research: CR. 41(1): p. 83. 10.1186/s13046-022-02295-4 PMC8892808 35241126

[25] Greene J, Segaran A, Lord S. Targeting OXPHOS and the electron transport chain in cancer; Molecular and therapeutic implications. Semin Cancer Biol. 2022;86(Pt 2):851–9. 35122973 10.1016/j.semcancer.2022.02.002

[26] Cadassou O, Jordheim LP. OXPHOS inhibitors, metabolism and targeted therapies in cancer. Biochem Pharmacol. 2023;211:115531. 37019188 10.1016/j.bcp.2023.115531

[27] Wu Z, et al. OMA1 reprograms metabolism under hypoxia to promote colorectal cancer development. EMBO Rep. 2021;22(1):e50827. 33314701 10.15252/embr.202050827 PMC7788456

[28] Ren L, et al. PHB2 promotes colorectal cancer cell proliferation and tumorigenesis through NDUFS1-mediated oxidative phosphorylation. Cell Death Dis. 2023;14(1):44. 36658121 10.1038/s41419-023-05575-9 PMC9852476

[29] Sriramkumar S, et al. Platinum-induced mitochondrial OXPHOS contributes to cancer stem cell enrichment in ovarian cancer. J Transl Med. 2022;20(1):246. 35641987 10.1186/s12967-022-03447-y PMC9153190

[30] Xu Y, et al. Why all the fuss about oxidative phosphorylation (OXPHOS)? J Med Chem. 2020;63(23):14276–307. 33103432 10.1021/acs.jmedchem.0c01013 PMC9298160

[31] Shao X, et al. The palmitoyltransferase ZDHHC21 regulates oxidative phosphorylation to induce differentiation block and stemness in AML. Blood. 2023;142(4):365–81. 37216691 10.1182/blood.2022019056

[32] Kalyanaraman B, et al. OXPHOS-targeting drugs in oncology: new perspectives. Expert Opin Ther Targets. 2023;27(10):939–52. 37736880 10.1080/14728222.2023.2261631 PMC11034819

[33] Li Y, et al. PINK1-Mediated Mitophagy promotes oxidative phosphorylation and Redox Homeostasis to Induce Drug-Tolerant Persister Cancer cells. Cancer Res. 2023;83(3):398–413. 36480196 10.1158/0008-5472.CAN-22-2370

[34] Sheryazdanova A, et al. The deubiquitinase OTUB1 governs lung cancer cell fitness by modulating proteostasis of OXPHOS proteins. Biochim Biophys Acta Mol Basis Dis. 2023;1869(7):166767. 37245529 10.1016/j.bbadis.2023.166767

[35] Deng J, et al. Exosomal transfer leads to chemoresistance through oxidative phosphorylation-mediated stemness phenotype in colorectal cancer. Theranostics. 2023;13(14):5057–74. 37771767 10.7150/thno.84937 PMC10526671

[36] Naldini MM, et al. Longitudinal single-cell profiling of chemotherapy response in acute myeloid leukemia. Nat Commun. 2023;14(1):1285. 36890137 10.1038/s41467-023-36969-0 PMC9995364

