# Supplementary figures and images for: Identification of TEFM as a potential therapeutic target for LUAD treatment
Source: J Transl Med. 2024 Jul 29;22:692. doi: 10.1186/s12967-024-05483-2 (PMC11288054; doi:10.1186/s12967-024-05483-2)

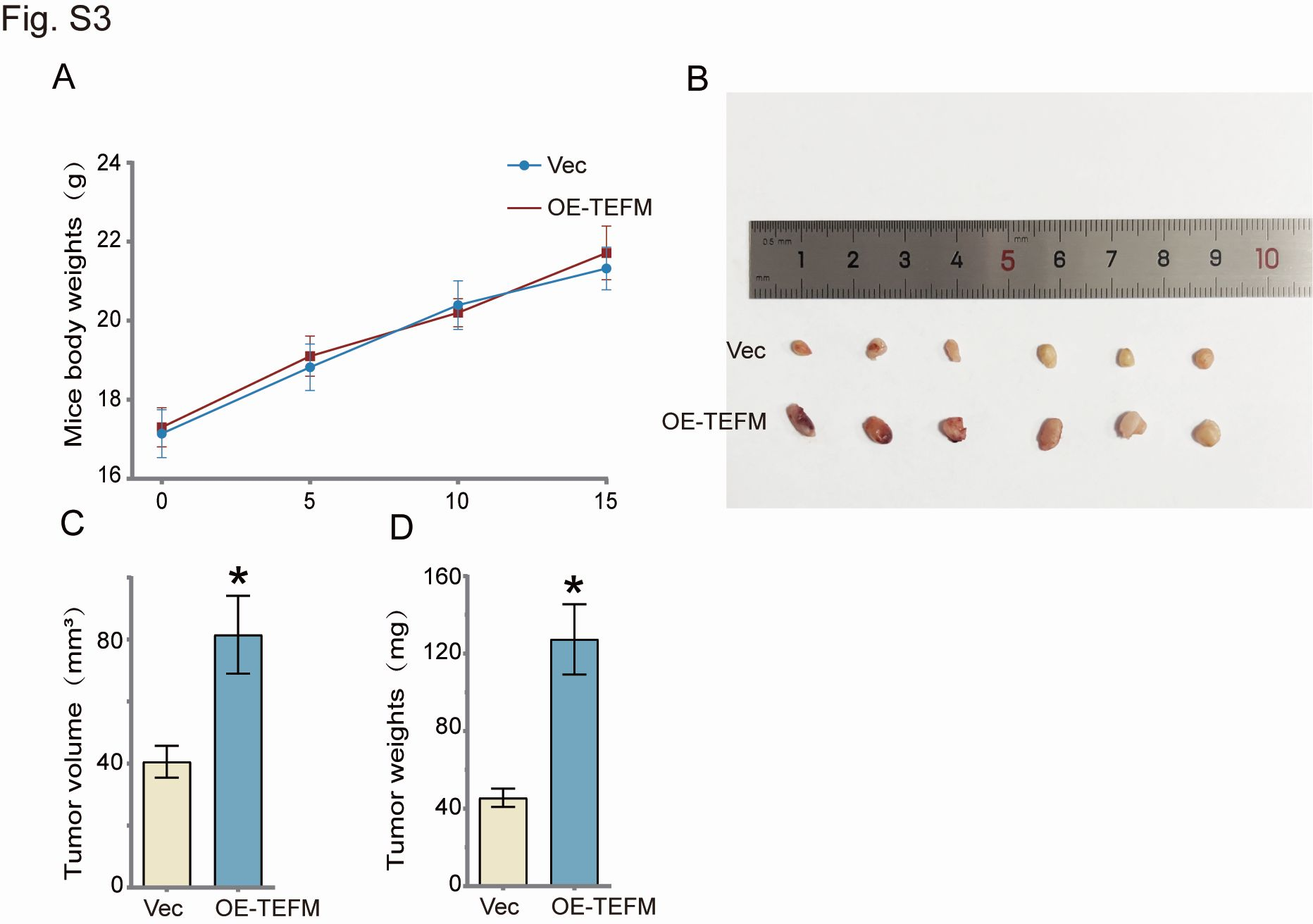

Supplement: Supplementary file 1 — Supplementary Material 1 [file 12967_2024_5483_MOESM1_ESM.png]

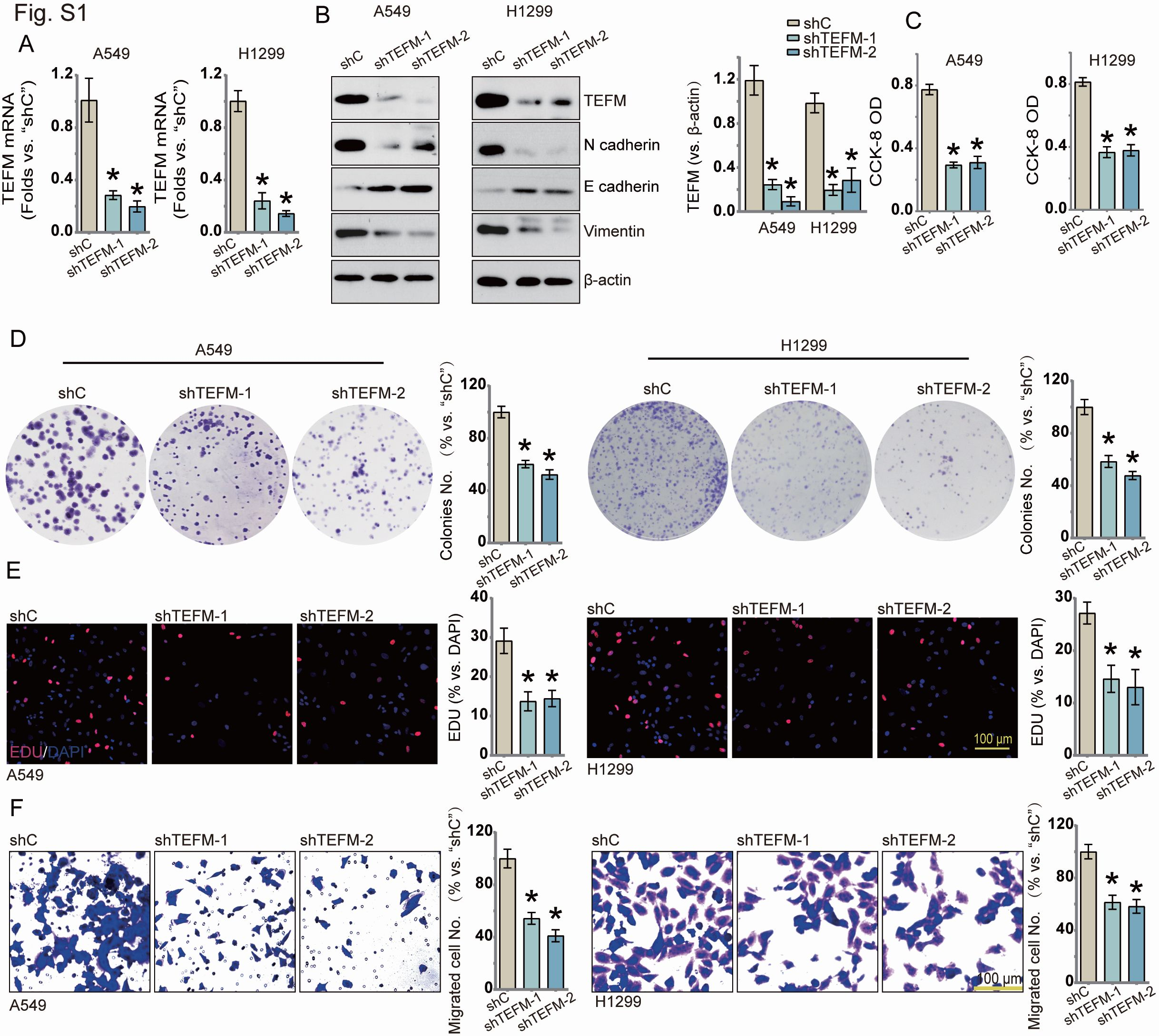

Supplement: Supplementary file 2 — Supplementary Material 2 [file 12967_2024_5483_MOESM2_ESM.png]

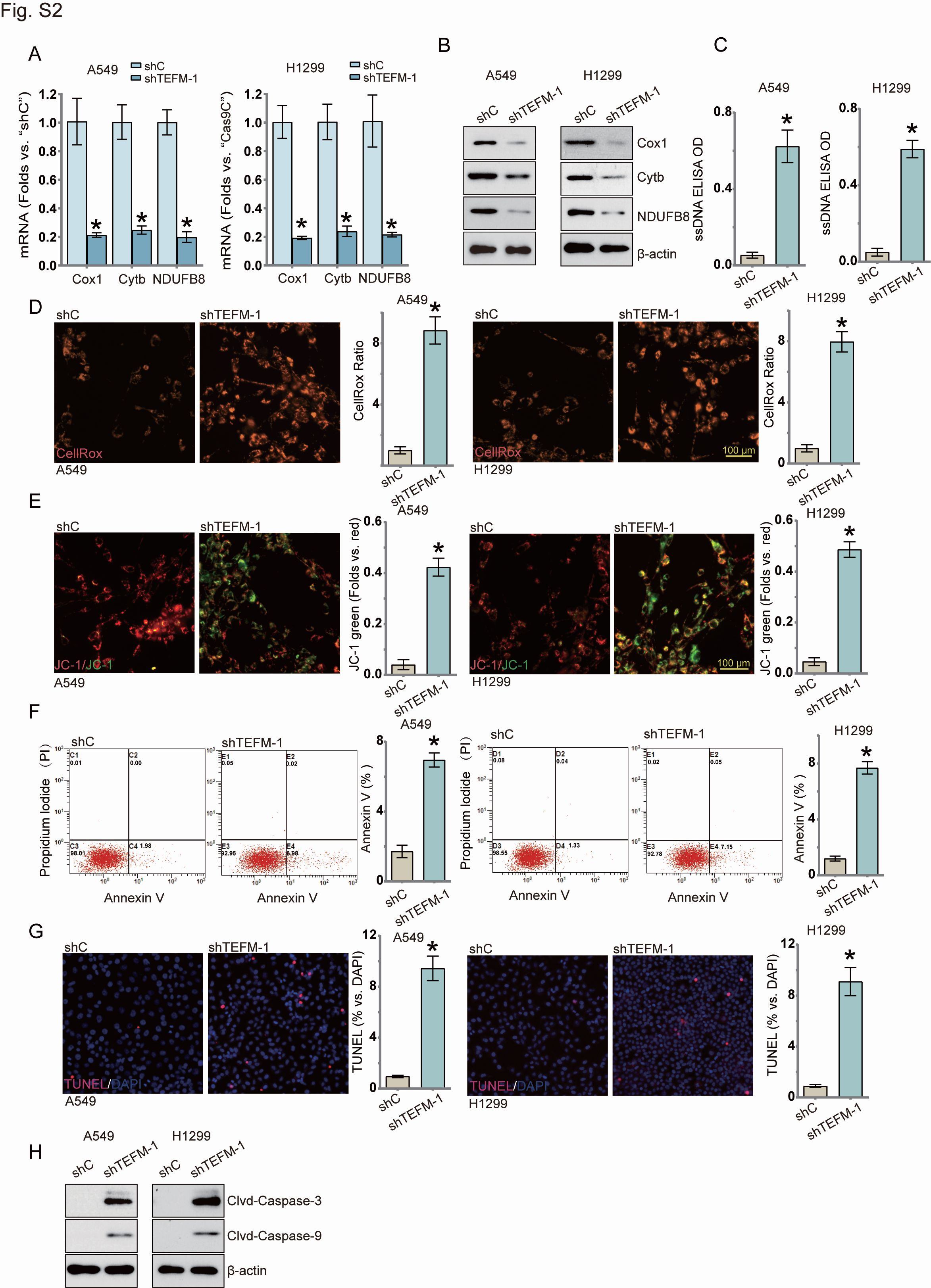

Supplement: Supplementary file 3 — Supplementary Material 3 [file 12967_2024_5483_MOESM3_ESM.png]
